# Long-term efficacy of allergen specific immunotherapy in atopic dermatitis patients

**DOI:** 10.1186/2045-7022-5-S1-P6

**Published:** 2015-03-11

**Authors:** Nina Wyrzykowska, Magdalena Czarnecka-Operacz

**Affiliations:** 1 Department of Dermatology, Poznañ University of Medical Sciences, Poznañ, Poland; 2 H. wiêcicki Hospital of Poznañ University of Medical Sciences, Department of Dermatology, Poznañ, Poland

## Background

Atopic dermatitis (AD) is an inflammatory, chronically relapsing, and highly pruritic skin disorder that considerably effects patients' life. As the aeroallergens are relevant eliciting factors of not only respiratory allergy but also AD, allergen-specific immunotherapy (ASIT) seems to be a curative therapy option in treating AD. The efficacy of ASIT in AD patients has been poorly investigated but the result are promising. We present the pioneer study on the long-term efficacy of ASIT in AD. The actual ASIT evaluation was performed after the observational period of 2-12 years after the termination of the treatment with allergy vaccines.

## Method

Fifteen patients suffering from AD, allergic to house dust mites or grass pollen allergens, who were previously treated with ASIT participated in the study. The analysis was performed based on selected clinical and laboratory parameters, quality of life and need for medication. The actual results were compared to those before ASIT was introduced as well as with those after its termination. To evaluate the skin condition W-AZS index was used. We investigated allergy parameters (total IgE and specific IgE) and immunological parameters (IL-2R, IL-4, IL-5, IL-10, INF- γ and ECP) .The quality of life evaluation was based on DLQI questionnaire. The need for antihistamine drugs, topical glicocorticosteroids as well as emollients was assessed.

## Results

Correctly performed ASIT in AD patients lead to quality of life improvement that persists even after 2-12 years after the termination of treatment. The improved quality of life involves everyday life comfort, social and professional life. ASIT improves skin condition, reduces pruritus during the treatment and,what is important, it leads to continuing long-term improvement after its termination. As a result the need for antihistamine drugs,topical glicocorticosteroids as well as emollients reduces and it persists at a constant level for years. ASIT does not affect the total and specific IgE at any level of observation. In a long term evaluation ASIT supresses a Th2 respond (IL-4,Il-5,IL-10) as well as Th1 respond (IL-2R,INF-γ) although the statistical analysis does not always reveal the significant difference. The ECP reduction confirms the anti-inflammatory effect of ASIT.

## Conclusion

This study confirms the effectiveness of ASIT in AD patients and it discloses the persistence of its results in long-term aspect.

